# Single‐center experience of ultra‐high‐density mapping guided catheter ablation of focal atrial tachycardia

**DOI:** 10.1002/clc.23774

**Published:** 2022-01-12

**Authors:** Antonia Kellnar, Stephanie Fichtner, Michael Mehr, Thomas Czermak, Moritz F. Sinner, Korbinian Lackermair, Heidi L. Estner

**Affiliations:** ^1^ Department of Medicine I University Hospital Munich, Ludwig Maximilians University Munich Germany; ^2^ German Cardiovascular Research Centre (DZHK), partner site: Munich Heart Alliance Munich Germany

**Keywords:** 3D mapping, ablation, atrial tachycardia, high density, Orion, rhythmia

## Abstract

**Introduction:**

Catheter ablation is the treatment of choice for recurrent focal atrial tachycardia (FAT) as medical therapy is limited. Routinely, a three‐dimensional mapping system is used. Whether or not optimized signal detection does improve ablation success rates has not yet been investigated. This retrospective cohort study compared ablation procedures using an ultra‐high‐density mapping system (UHDM, Rhythmia, Boston Scientific) with improved signal detection and automatic annotation with procedures using a conventional electroanatomic mapping system (CEAM, Biosense Webster, CARTO).

**Methods:**

All patients undergoing ablation for FAT using UHDM or CEAM from April 2015 to August 2018 were included. Endpoints comprised procedural parameters, acute success as well as freedom from arrhythmia 12 months after ablation.

**Results:**

A total of 70 patients underwent ablation (48 with UHDM, 22 with CEAM). No significant differences were noted for parameters like procedural and radiation duration, area dose, and RF applications. Acute success was significantly higher in the UHDM cohort (89.6% vs. 68.2%, *p* = .03). Nevertheless, arrhythmia freedom 12 months after ablation was almost identical (56.8% vs. 60%, *p* = .87), as more patients with acute success of ablation presented with a relapse during follow‐up (35.0 vs. 7.7%, *p* = .05).

**Conclusion:**

Acute success rate of FAT ablation might be improved by UHDM, without an adverse effect on procedural parameters. Nevertheless, further research is needed to understand the underlying mechanism for increased recurrence rates after acute successful ablation.

## 1 INTRODUCTION

Focal atrial tachycardia (FAT) is defined as organized atrial rhythm >100 beats per minute initiated from a discrete origin and spreading over both atria in a centrifugal pattern.^1^, ^2^ The arrhythmia may be sustained or incessant. Dynamic forms with recurrent interruptions and reinitiations may be frequent.^1^ Patients suffering from recurrent FAT are often highly symptomatic and uncontrolled FAT can cause tachymyopathic impairment of left ventricular function.^3^ Efficacy of medical treatment is limited, whereas catheter ablation is a feasible and successful therapeutic option for symptomatic FAT.^4^ Thus, the current ESC guideline prefers catheter ablation of chronic FAT especially if incessant or causing tachycardiomyopathy instead of antiarrhythmic treatment (recommendation class I).^1^

With the increasing importance of catheter ablation for the treatment of FAT, long term success‐rates have been published. In previous procedures conventional mapping technique was utilized with acute success rates varying from 60% to 70%, but with the implementation of electroanatomical mapping (EAM) even higher success rates could be achieved (80%–90%).^4^, ^5^, ^6^, ^7^ Still, mapping the tachycardia remains challenging with established EAM systems, for example, CARTO or Ensite NavX, as frequent extensive and accurate mapping is required for identifying the origin of the tachycardia.^4^, ^6^, ^8^, ^9^ The ultra‐high density mapping (UHDM) system (Rhythmia, Boston Scientific) provides increasingly growing and promising data in RFA of common rhythm disorders. In several studies, UHDM was fast and feasible without compromising safety criteria.^10^, ^11^, ^12^, ^13^ Successful mapping of complex macro‐ and micro‐reentrant atrial tachycardia were reported especially in patients with a history of previously failed ablations using conventional mapping systems.^11^, ^13^, ^14^, ^15^ In one single center study FAT seems to benefit from using UHDM for ablation of FAT, but additional data, especially about long‐term outcome is lacking.^10^

The aim of our study was to compare ablation procedures using UHDM versus CEAM with respect to acute success of ablation as well as 12 months outcome in an all‐comer collective of consecutive patients undergoing catheter ablation of symptomatic FAT in a predefined time period.

## 2 METHODS

### 2.1 Patient population

From April 2015 to August 2018 all patients undergoing catheter ablation and intraprocedural documented FAT at the Department of Medicine I, University Hospital Munich, Ludwig‐Maximilians Universitiy, Munich, Germany were included in our retrospective trial and studied separately in two groups (UHDM and CEAM). During this period each mapping system was deployed in a fixed agenda on predefined days. Clinical and periprocedural data was collected from the electronical medical records and the data base of the electrophysiology laboratory. A follow‐up was performed either by inpatient or outpatient planned visit or by phone with no prescheduled follow‐up period. Sinus rhythm during 12‐lead‐ECG and Holter‐monitoring combined with freedom from symptoms like palpitations, symptomatic rapid heartbeat was determined as a success. Any symptomatic supraventricular tachycardia (with or without ECG documentation) that occurred during the follow‐up period was defined as a relapse. Any patient with ablation failure was classified as a “recurrence” for 12‐month follow‐up without new evaluation. During this retrospective analysis no additional examinations or treatments were conducted beyond routine clinical care. The investigation was approved by the local Ethics Committee of the Ludwig‐Maximilians‐University of Munich and performed in accordance with the declaration of Helsinki. Because of the retrospective nature of our study, informed consent was waived by the approving ethics committee.

### 2.2 Procedural protocol

Six operators conducted the ablation procedures. The procedure was undertaken under local anesthesia and conscious sedation with midazolam and remifenantil. Venous access was accomplished with sheaths (6–11 Fr.) in the femoral veins. Where necessary, hemodynamic monitoring was obtained via the right radial (4 Fr.) or for catheter placement via the femoral artery (8–9 Fr.). Routinely, a 6 Fr. steerable decapolar catheter was placed in the coronary sinus. If needed, a transseptal punction for accessing the left atrium was performed using a deflectable sheath (Agilis, Abbott) under hemodynamic and fluoroscopic monitoring. Each procedure was conducted in accordance with a standardized protocol: Orciprenalin was applied in case of insufficient spontaneous or electrical induction. Discrimination of triggered activity and micro‐reentrant tachycardia mechanism remains challenging even in ablation procedures using high‐density mapping. In our study, an arrhythmia was diagnosed as focal AT in presence of centrifugal activation, cycle length variation, and inconsistent entrainment responses and in absence of visualization of a re‐entry in LAT mapping. Radiofrequency was applied for at least 30 s, if unsuccessful it was stopped. With successful ablation duration was up to the operator's decision. For irrigated ablation with CARTO, 30 W and an irrigation rate for 30 ml/min were applied. In the UHD group a surround flow catheter with an irrigation rate of 8 ml/min and a power of 30 W was used. After initial termination, a 30 min waiting period was routinely implemented. Acute procedural success was achieved if the tachycardia was non‐inducible, not only spontaneously but also by burst‐stimulation, extrastimuli or orciprenalin application for at least 30 min after the last energy application.

#### 2.2.1 UHDM

The Rhythmia mapping system combines magnetic and impedance‐based localization for accurate electroanatomic mapping. A magnetic back patch and a stationary reference electrode (conventionally placed in the coronary sinus) built the location reference. The mapping catheter Intella‐Map‐Orion is constructed with 64 flat microelectrodes (diameter 0.8 mm) and 2.5 mm interelectrode distance in a basket formation with 8 splines (8.5 F). The basket configuration enables bidirectional flexion and variable levels of deployment with ranges from 3 to 22 mm, which enables individual adjustment in different anatomic conditions. The system is based on three fundamental features: 1. High density guaranteed by the 64 electrodes of the Intella‐Map‐Orion catheter, which creates a higher point density than comparable mapping systems, and the possibility to acquire an unlimited number of EGMs. 2. High resolution due to a noise floor that enables visualization of low voltage areas. 3. Fast acquisition and automatic annotation of thousands of electrograms by validating each continuously acquired point based on predefined criteria for beat acceptance length: relative timing of the reference electrode, synchronization of respiratory cycles, the pattern of the catheter movement, and the tracking quality. UHMD does not limit the selection of the ablation catheter and enables RFA as well as cryoablation.

#### 2.2.2 CEAM

FAT ablation procedures performed with the CARTO mapping system during the same time period were enrolled as a control cohort. This 3D electroanatomic mapping system (Biosense Webster) is widely used for mapping common rhythm disorders and complex arrhythmias. Three coils placed in a locator pad generate a low‐level magnetic field and the magnetic power of each coil is measured by a sensor located at the tip of the mapping catheter. For creation of 3D Map the respective ablation catheter or a multi‐electrode mapping catheter with 20 (CARTO Lasso) or 22 (CARTO Pentaray) electrodes was employed. By combining the strength of each coil and transforming the results into distance parameters, three‐dimensional geometry of the chamber and the local activation times can be generated. Radiofrequency was applied using the ThermoCool SmartTouch catheter (Biosense Webster) and catheter stability during RF application was controlled by measuring contact force (CF).^16^

### 2.3 Statistics

For continuous variables all data is described as a median ± standard deviation (SD). Nominal variables are analysed using the *χ* ^2^ test, continuous variables were compared by the Median test. A Kaplan–Meier curve was used to show recurrence rates graphically, comparisons were made by the Log‐rank test. A two‐tailed *p* ≤ .05 was rated as statistically significant. All statistical analyses were conducted using SPSS version 25 (IBM Corp.).

## 3 RESULTS

### 3.1 Patient characteristics

From April 2015 to August 2018, 48 patients underwent ablation for FAT with UHDM and 22 with CEAM. Baseline characteristics are presented in Table 1.

**Table 1:** Baseline characteristics

	All patients	UHD‐mapping	CEA‐mapping	*p*
Patients, *n* (%)	70	48 (68.6%)	22 (31.4%)	
Age (years)	62.3 (50.3; 74.7)	56.9 (49.2; 72.3)	71.8 (49.8; 72.3)	.07
Female sex, *n* (%)	37 (52.9%)	26 (54.2%)	11 (50.0%)	.75
Previous FAT‐ablation, *n* (%)	10 (14.3%)	8 (16.7%)	2 (9.1%)	.64
Risk factors				
Diabetes, *n* (%)	11 (16.7%)	5 (11.4%)	6 (27.3%)	.10
Hypertension, *n* (%)	38 (58.5%)	22 (51.2%)	16 (72.7%)	.10
Dyslipidemia, *n* (%)	26 (40.0%)	15 (34.9%)	11 (50.0%)	.24
CHA_2_DS_2_‐VASc	2 (1; 4)	2 (1;3)	3 (2;5)	**.01**
HAS‐BLED	2 (1;2)	2 (1;2)	2 (1;3)	.39
Creatinine (mg/dl)	1.0 (0.9; 1.11)	1.0 (0.9; 1.1)	1.0 (0.9; 1.3)	.65
NT‐proBNP (pg/ml)	383.0 (113; 955)	424 (111; 1042)	198 (121; 954)	.73
Comorbidities				
Coronary artery disease, *n* (%)	19 (27.1%)	8 (16.7%)	11 (50.0%)	**.01**
MI, *n* (%)	2 (2.9%)	2 (4.2%)	0 (0%)	.33
COPD, *n* (%)	3 (4.3%)	2 (4.2%)	1 (4.5%)	.94
Cardiomyopathy, *n* (%)	24 (34.3%)	17 (35.4%)	7 (31.8%)	.77
Valvular heart disease, *n* (%)	11 (15.7%)	8 (16.7%)	3 (13.6%)	.75
Cardiac surgery, *n* (%)	5 (7.1%)	3 (6.3%)	2 (9.1%)	.67
Pacer, *n* (%)	4 (5.7%)	2 (4.2%)	2 (9.1%)	.41
ICD, *n* (%)	9 (12.9%)	7 (14.6%)	2 (9.1%)	.52
Medication				
Antiarrhythmic therapy, *n* (%)	64 (91.4%)	42 (87.5%)	22 (100%)	.08
Betablocker, *n* (%)	60 (85.7%)	39 (81.3%)	21 (95.5%)	.12
Class‐I‐antiarrhythmic drugs, *n* (%)	4 (5.7%)	2 (4.2%)	2 (9.1%)	.41
Class‐III‐antiarrhythmic drugs, *n* (%)	10 (14.3%)	5 (10.4%)	5 (22.7%)	.17
Class‐IV‐antiarrhythmic drugs, *n* (%)	11 (15.7%)	8 (16.7%)	3 (13.6%)	.75
Cardiac glycosids, *n* (%)	3 (4.3%)	1 (2.1%)	2 (9.1%)	.18

Abbreviations: CEA, conventional electroanatomic; COPD, chronic obstructive pulmonary disease; ICD, implantable cardioverter‐defibrillator; MI, myocardial infarction; TIA, transient ischemic attack; UHD, ultrahigh‐density.

### 3.2 Procedural characteristics and endpoints

**Figure 1:**
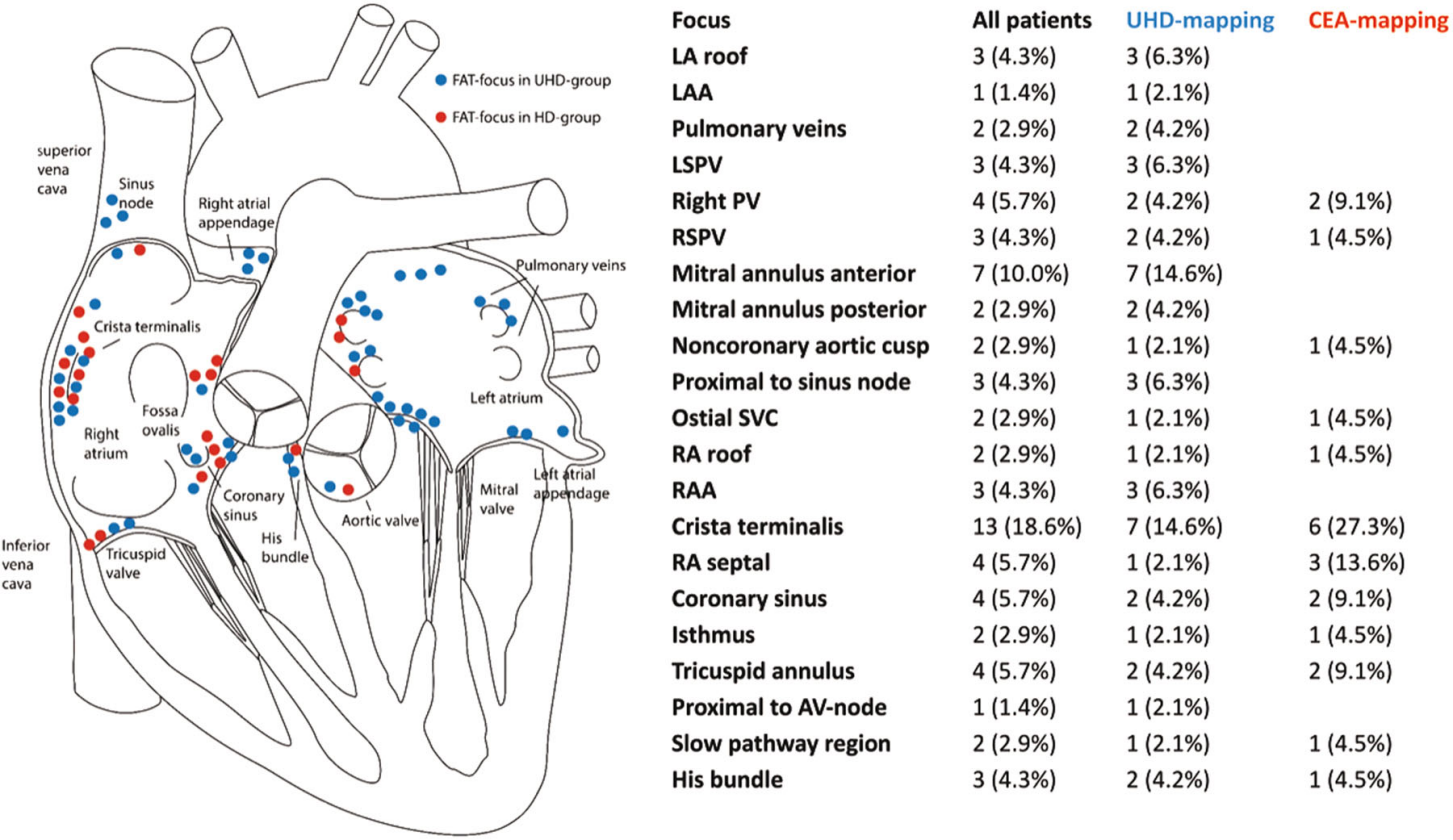
Intraprocedural focus of the focal atrial tachycardia. CEA, conventional electroanatomic; LA, left atrium; LAA, left atrial appendage; LSPV, left superior pulmonary vein; PV, pulmonary vein; RA, right atrium; RAA, right atrial appendage; RSPV, right superior pulmonary vein; SVC, superior vena cava; UHD, ultrahigh‐density

Figure 1 depicts the distribution pattern of the respective focus of the tachycardia graphically and procedural endpoints are listed in Table 2. In the UHD group, all maps were created using the Orion mapping catheter. Multi‐electrode mapping catheters were used in 45% of all CEAM procedures, with the remaining maps created using the ablation catheter (Table S1). Accordingly, EGM count differed significantly.

**Table 2:** Periprocedural data

Periprocedural data							
	All patients		UHD‐mapping		CEA‐mapping		*p*
Origin of FAT	LA	24 (34.3%)	LA	22 (45.8%)	LA	2 (9.1%)	.02
	RA	41 (58.6%)	RA	24 (50.0%)	RA	17 (77.3%)	
	BA	3 (4.3%)	BA	1 (2.1%)	BA	2 (9.1%)	
	AV	2 (2.9%)	AV	1 (2.1%)	AV	1 (4.5%)	
Ablation catheter							
Standard tip	6 (8.6%)		7 (14.6%)		0		.08
Contact force	23 (32.9%)		1 (2.1%)		22 (100%)		<.01
Open irrigated	60 (85.7%)		38 (79.2%)		22 (100%)		.02
Cryo	2 (2.9%)		2 (4.2%)		0		.33
Acute Success:							
All patients, *n* (%)	58 (82.9%)		43 (89.6%)		15 (68.2%)		**.03**
Patients with previous FAT‐ablation, *n* (%)	10 (100%)		8 (100%)		2 (100%)		**‐**
Procedural failure:							
Mapping failure	4 (5.7%)		1 (2%)		3 (13.6%)		.13
Withheld of ablation	4 (5.7%)		2 (4.2%)		2 (9.1%)		.46
No monofocal AT inducible	4 (5.7%)		2 (4.2%)		2 (9.1%)		.46
Procedural time (min)	180 (119; 219)		175 (123; 215)		190 (106; 224)		.63
Radiation time (min)	8.0 (5.0; 15.1)		8.6 (5.1; 15.4)		6.7 (3.5; 10.4)		.40
Contrast agent (ml)	0 (0;0)		0 (0;0)		0 (0; 18.8)		.87
Area dose (Gy*cm²)	186.0 (91.5; 394.5)		206.0 (80.0; 383.0)		151.5 (99.5; 681.8)		.36
EGMs (*n*)	4075 (697; 6439)		5456 (3364; 7913)		247 (139; 859)		**<.01**
AT cycle length (ms)	391.5 (319.0; 485.5)		385.0 (312.0; 465.0)		443.5 (337.3; 526.8)		.41
local voltage at successful ablation site (mV)	0.58 (0.30; 1.22)		0.54 (0.27; 1.13)		0.87 (0.50; 1.58)		.67
Duration of signal (ms)	34.0 (29.0; 44.5)		37.0 (29.3; 49.0)		33.0 (26.5; 35.8)		.07
Mapping volume (cm²)	90.8 (67.1; 116.5)		91.2 (64.2; 121.4)		86.3 (68.9; 113.9)		.93
Mapping duration (min:s)	18:19 (10:58; 31:00)		15:55 (09:10; 24:05)		40:00 (16:45; 126:15)		**.04**
RF applications (*n*)	2.5 (5.0; 13.5)		5.0 (2.0; 14.5)		8.0 (3.0; 13.0)		.35
RF application duration (s)	256.0 (120.0; 785.0)		403.0 (120.0; 742.5)		180.0 (130.0; 1005.0)		.45
Mean RF duration per RF application	45.0 (30.1; 82.8)		52.0 (30.2; 87.7)		37.3 (21.7; 64.6)		.66
Complications, *n* (%)	3 (4.3%)		1 (2.1%)		2 (9.1%)		.18
Minor groin bleeding, *n* (%)	1 (1.4%)		1 (2.1%)		‐		
Major groin bleeding, *n* (%)	1 (1.4%)		‐		1 (4.5%)		
Pacer implantation, *n* (%)	1 (1.4%)		‐		1 (4.5%)		

*Note*: The values in bold reach the significance level of *p* <= 0.05.

Abbreviations: AT, atrial tachycardia; AV, aortic valve; BAA, biatrial; CEA, conventional electroanatomic; EGM, electrogram; FAT, focal atrial tachycardia; LA, left atrium; RA, right atrium; RF, radiofrequency; UHD, ultra‐high‐density.

Each ablation in the CEAM group utilized the irrigated Thermocool Smarttouch SF ablation catheter. Different irrigated and standard tip RF catheters and also cryo catheters were used in the UHD group (Table S1).

Acute ablation success was achieved in 89.6% of patients treated with the UHD‐mapping system and in 68.2% of patients in the control cohort (*p* = .03). Overall, de‐escalation of antiarrhythmic pretreatment was possible in 5 (8.6%).

Acute success rates in the control group did not differ between procedures using multielectrode mapping catheters and procedures mapping with the ablation catheter (50% vs. 83%; *p* = .10, Table S1). This observation did not reach statistical significance.

The reasons for unsuccessful ablation procedures are listed in Table 2. Ablation failure as a consequence of mapping failure was by trend more frequent in the CEAM group (13.6. vs. 2%; *p* = .13).

Map creation was significantly faster in the UHD group (mapping time 15:55 min vs. 40 min, *p* = .03). No relevant differences were noted for procedural duration, radiation time, and dose. Ablations with CEAM required a higher number of RF applications (8 vs. 5, *p* = .34) with equal mean RF duration per RF application (52 vs. 37.3 s, *p* = .65). Procedural parameters are illustrated in Figure S2.

### 3.3 Clinical outcome

A 12‐month follow‐up was available for 92.9% of all patients. The overall recurrence rate was 42.2%. As a consequence, antiarrhythmic therapy was escalated within the 12 months after ablation in two patients of the UHDM group (Figure S1).

**Figure 2:**
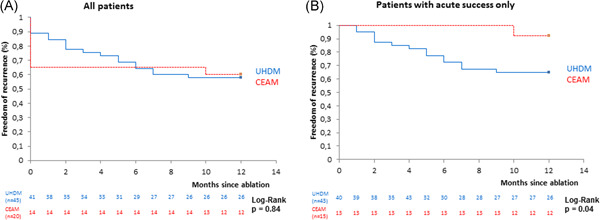
Freedom of arrhythmia recurrence 12 months after ablation. CEAM is depicted in red, UHDM in blue lines. CEAM, conventional electroanatomic mapping system; UHDM, ultra‐high‐density mapping system

No relevant difference was seen between UHDM and CEAM (43.2 vs. 40%, *p* = .87, Table 3a, Figure 2A), even when separately analysed with regard to the focus origin: FAT with left atrial focus was recurrent in seven patients in the UHD‐group versus one patient in the CEA‐group (36.8% vs. 33.3%, *p* = .29), with right atrial focus recurrence was documented in 11 patients in the UHD‐group and seven patients in the control cohort (45.8% vs. 46.7%, *p* = .96).

**Table 3:** Clinical outcome

	All patients	UHD‐mapping	CEA‐mapping	*p*
(a) Follow up (all patients)				
FU completed, *n* (%)	64 (91.4%)	44 (91.7%)	20 (90.9%)	.92
Recurrence after 12 months, *n* (%)	27 (42.2%)	19 (43.2%)	8 (40%)	.87
(b) Follow up (patients with acute success only)				
FU completed, *n* (%)	53 (91.4%)	40 (93.0%)	13 (86.7%)	.45
Recurrence after 12 months, *n* (%)	15 (28.3%)	14 (35.0%)	1 (7.7%)	**.05**
(c) Follow up (*1st Do* ablations only)				
FU completed, *n* (%)	33 (91.7%)	25 (92.6%)	8 (88.9%)	.73
Recurrence after 12 months, *n* (%)	7 (21.2%)	7 (28.0%)	0	.09

*Note*: The values in bold reach the significance level of *p* <= 0.05.

Abbreviations: CEA, conventional electroanatomic; FU, follow up; UHD, ultra‐high‐density.

A significant numerical difference of recurrence rates was seen in patients with acute successful ablation. 35.0% of the UHDM and 7.7% of patients in the CEA cohort showed recurrence within the first 12 months after successful ablation (*p* = .05, Table 3b, Figure 2B). For *1st do* ablations only we did not find any significant differences in 12‐month recurrence (Table 3c).

### 3.4 Safety

The overall periprocedural complication rate was 4.3%, fewer in number in the UHDM group (2.1% vs. 9.1%) with no significant difference (Table 2). No cerebrovascular events or pericardial tamponades were observed. One case of minor groin bleeding was noted in the UHDM group. In the control cohort one patient required a postprocedural pacemaker implantation after ablation proximal to the AV‐node and one patient was suffering from a major groin bleeding, which necessitated a blood transfusion. No bleeding event required interventional or surgical treatment. None of the described complications were attributed to the utilized mapping system.

## 4 DISCUSSION

Catheter ablation has become the therapy of choice for (mono‐) focal atrial tachycardia. To the best of our knowledge this is the first report that allows a direct comparison of acute ablation success rates and 12 months clinical outcome after ablation utilizing UHMD or CEAM in a retrospective analysis of a single‐center all comer cohort.

The major finding of our study was the improved acute ablation success in procedures using UHDM (89.6% vs. 68.2%, *p* = .03). This advantage did not convert into an improvement of arrhythmia freedom after 12 months as more patients with acute successful ablation showed a relapse during follow‐up (35 vs. 7.7%, *n* = 14 vs. *n* = 1, *p* = .05).

In a large prospective cohort study among 431 patients undergoing ablation procedures for FAT using conventional or HD‐mapping systems, an acute success rate of 84% was documented. 4.8% of patients had an early recurrence and 19% had a late recurrence during the observation period of 12 months.^6^ As ablation procedures and mapping techniques evolved, benefits of EAM over conventional mapping systems were noted, in particular for complex cases or for those with recurrent tachycardia after a conventional mapping approach.^4^, ^6^, ^8^, ^17^, ^18^

With rhythmia a UHDM system was established, which provides fast acquisition of a significantly higher amount of EGMs due to high electrode density. The innovative shape and construction of the mini‐basket mapping catheter with 64 closely arranged microelectrodes enables high resolution with superior density of electrograms and allows both, acquisition of activation timing and voltage data in a short time. After initial experiences with UHDM in animal models, several studies have reported feasibility, safety and also beneficial effects on a variety of radiofrequency ablation procedures for a variety of rhythm disorders.^10^, ^11^, ^12^, ^13^, ^15^, ^19^, ^20^ Comparison of UHDM and CEAM for first Do PVI procedures could demonstrate a more than fivefold increased EGM density in operators familiar with UHDM.^10^ Map creation with UHDM mapping was significantly faster compared to the CEAM group in our study. Due to higher electrode density on the Orion mapping catheter, which allows fast acquisition of a high amount of EGMs, information is gathered more quickly using the UHD mapping system. Additionally, the valid automated annotation algorithm produces to reliable maps without the need for manual reannotation or remapping. The superior density can be crucial for activation mapping of FAT as conduction velocity adjacent to the origin of FAT is very high (as illustrated in Figure S1) and may serve as an explanation for the improved acute ablation success. Nevertheless, this possible superiority did not convert to a higher rate of 12 months arrhythmia freedom (recurrence after 12 months 43.2% vs. 40%).

Optimal tissue contact during RF application is crucial for optimal ablation efficacy and generation of durable lesions. In RF application using standard tip catheters, tip temperature gives sufficient contact feedback. This measure of tissue contact gets lost with catheter irrigation. A technical solution for this circumstance is the measurement of CF available in Smart Touch (Biosense Webster) or TactiCath (Abbot) ablation catheters. The ablation catheter used in UHD mapping were visualized only by impedance information. This might be a significant disadvantage compared to CARTO, where impedance, magnetic, and CF information improves the visualization of the mapping catheter.

The use of CF catheters in ablation of paroxysmal AF can improve freedom from arrhythmia recurrence, shorten procedural times as well as fluoroscopy and RF times^21^ and may also assist the creation of effective lesions in the context of scar‐related VT ablation.^22^

In our cohort, CF catheters were used in every ablation procedure using CEAM but only one procedure using UHDM. One could speculate, that problems in effective lesion creation might have led to a less durable lesion formation, which in turn might have led to an increase in recurrence rates after primarily successful ablation.

Our investigation contains major limitations. First, a matched control group with a similar sample size was lacking and the procedural strategy was not randomized, but chosen by the individual operator. This may bias our results, as a mapping system could be selected using the expected complexity as a criterion and could be reflected in the difference of patient numbers in the two cohorts. Second the inhomogeneity of utilized ablation catheters impedes comparability in our retrospective analysis significantly. Furthermore, to overcome underrated recurrence rates we extended our definition of recurrence to any symptomatic tachycardia during our follow‐up period. However, this approach could lead to overestimated results, especially in patients with multiple atrial tachycardia or a complex history of rhythm disorders.

## 5 CONCLUSION

Acute success rates of FAT ablation could be improved by UHDM, without an adverse effect on procedural parameters and safety. Nevertheless, further research is needed to understand the underlying mechanism of the increased recurrence rates after acute successful ablation. This study will require a multicentre randomized approach, not only for the mapping system but also for the selected ablation catheter.

## CONFLICTS OF INTERESTS

Heidi Estner has received honoraria for lectures from Boehringer Ingelheim, Boston Scientific, Medtronic and honoraria for advisory board activities from Boston Scientific.

## AUTHOR CONTRIBUTIONS

The contributing authors are Antonia Kellnar (conception and design, analysis and interpretation of data, drafting of the manuscript or revising it critically for important intellectual content), Stephanie Fichtner (conception and design or analysis and interpretation of date, final approval of the manuscript submitted), Michael Mehr (final approval of the manuscript submitted), Thomas Czermak (final approval of the manuscript submitted), Moritz F. Sinner (final approval of the manuscript submitted), Korbinian Lackermair (conception and design, analysis and interpretation of data, drafting of the manuscript or revising it critically for important intellectual content), and Heidi L. Estner (conception and design, analysis and interpretation of data, drafting of the manuscript or revising it critically for important intellectual content). All authors take responsibility for all aspects of the reliability and freedom from bias of the data presented and their discussed interpretation.

## Supporting information

Supporting information.

## Data Availability

The data underlying this article will be shared anonymized on reasonable request to the corresponding author.
