# Supplementary material for: Single‐center experience of ultra‐high‐density mapping guided catheter ablation of focal atrial tachycardia
Source: Clin Cardiol. 2022 Jan 12;45(3):291–8. doi: 10.1002/clc.23774 (PMC8922533; doi:10.1002/clc.23774)
Supplement: Supplementary file 1 — Supporting information. [file CLC-45-291-s001.docx]

**Single center experience of ultra high-density mapping guided catheter ablation of focal atrial tachycardia**

Antonia Kellnar, MD^1^, Stephanie Fichtner, MD^1^, Michael Mehr, MD^1^, Thomas Czermak, MD^1^, Moritz F. Sinner, MD, MPH^1,2^, Korbinian Lackermair, MD^1*^, Heidi L. Estner MD^1*^

^1^Department of Medicine I, University Hospital Munich, Ludwig Maximilians University, Munich, Germany

^2^German Cardiovascular Research Centre (DZHK), partner site: Munich Heart Alliance, Munich, Germany

*equal contribution

Supplement:


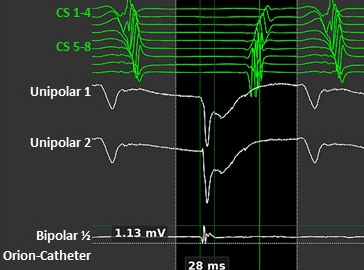
Supplemental Figure 1.
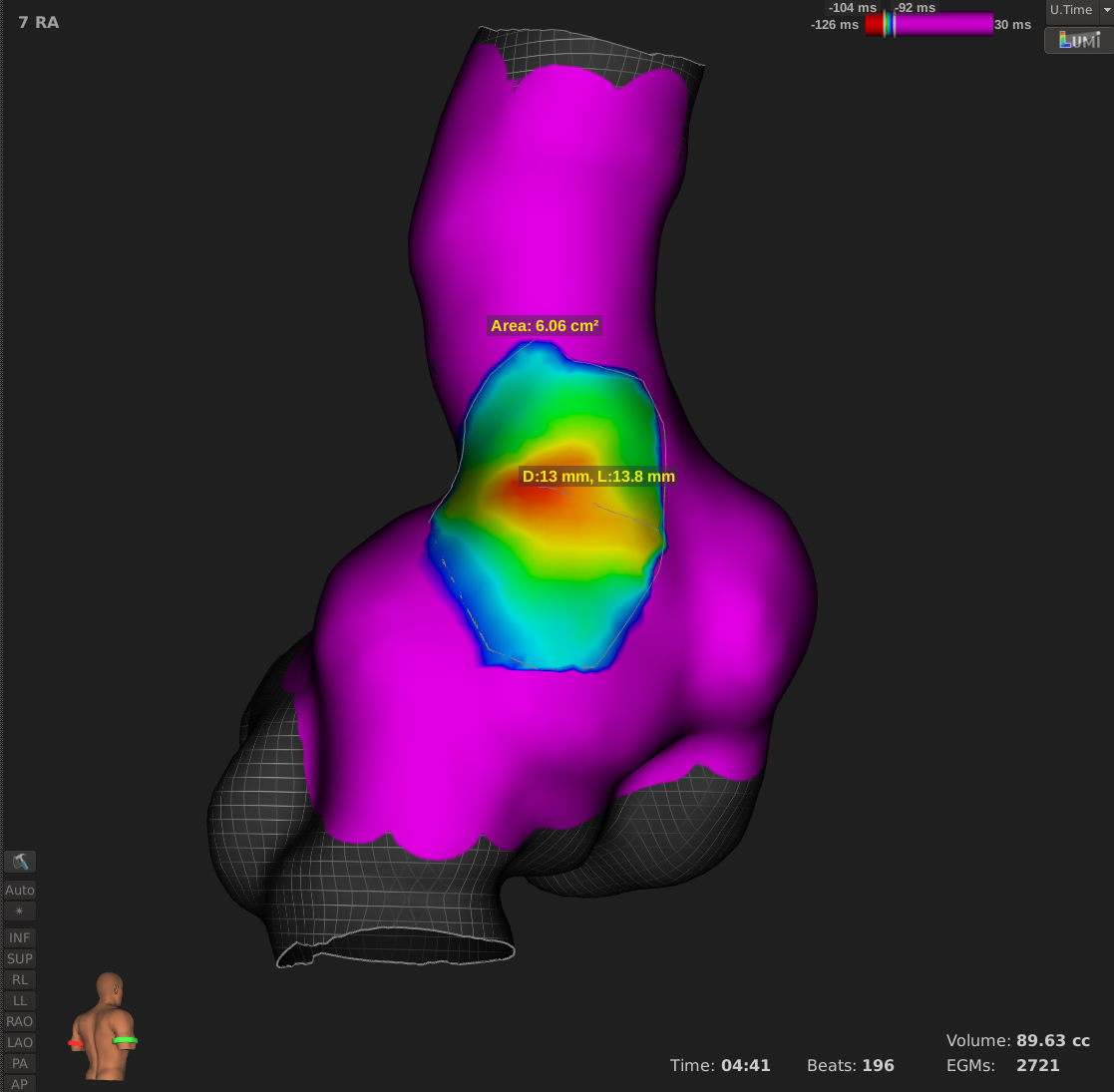


LAT Map of a sinus node near FAT. Right posterior oblique angulation. The EGM of the ablation focus is shown on the left, mapping time, acquired tachycardia beats as well as EGM count and mapping volume are shown in the right bottom line.

The focal origin is visualized in red colour and located posterior in the RA close to the insertion of superior vena cava. High conduction velocity in the present case with healthy myocardium results in an activated area of 6.06cm² within the first 10 ms after tachycardia onset (activation velocity: 2.65 m/s). This case illustrates the advantage of improved temporospatial resolution by UHD mapping.

|  | **UHD-mapping** | | | **CEA-mapping** | |
| --- | --- | --- | --- | --- | --- |
| **Mapping catheter** | | | | | |
| Multielectrode catheter | Orion | | 48 (100%) | Lasso  Pentaray | 6 (27.3%)  4 (18.2%) |
| Ablation catheter | - | | | Thermocool Smarttouch | 12 (54.5%) |
| **Acute success** | | | | | |
| Mapping via multi electrode catheter  Mapping via ablation catheter | 43 (89.6%)  - | | | 5 (50.0%)  p=0.10  10 (83.3%) | |
| **Ablation catheter** | | | | | |
|  | Thermocool SF MarinR MC  IntellaNAV MIFI Tacticath Quartz Biosense Celsius  Freezor Xtra | 29 (60.4%)  8 (16.6%)  6 (12.5%)  1 (2.1%)  1 (2.1%)  2 (4.2%) | | Thermocool Smarttouch | 22 (100%) |
| **Acute success** | | | | | |
|  | Thermocool SF  MarinR MC  IntellaNAV MIFI  Tacticath Quartz  Biosense Celsius  Freezor Xtra 2 | 26 (89.7%)  7 (87.5%)  6 (100%) p=0.13  1 (100%)  1 (2.1%)  2 (100%) | | Thermocool Smarttouch SF | 15 (68.2%) |

Supplemental table 1: Mapping and ablation catheters and catheter-dependent success rate.

Boston Scientific: Orion, IntellaNAV MIFI; Biosense Webster: Lasso, Pentaray, Thermocool Smarttouch SF, Thermocool SF, Biosense Celsius; Medtronic: MarinR MC, Freezor Xtra; Abbott: Tacticath Quartz

Supplemental Figure 2.


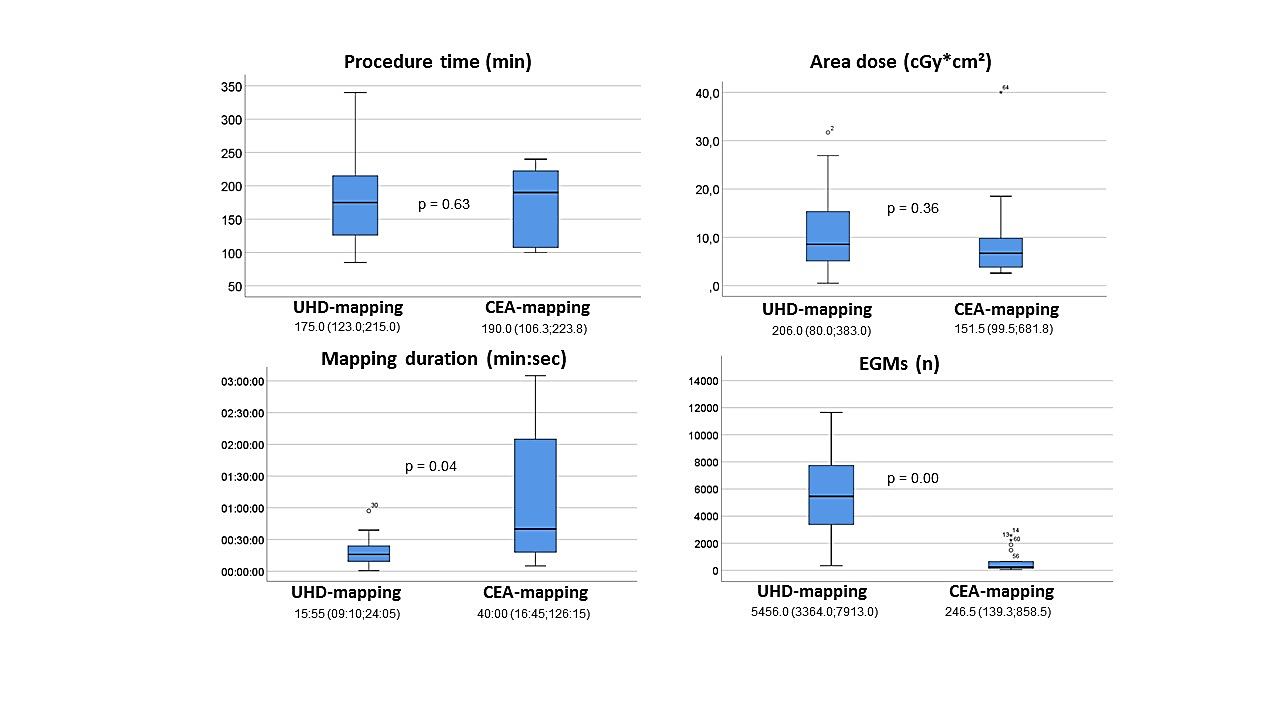


Periprocedural parameters.

UHD: ultra-high-density; CEA: conventional electroanatomical
